# Sanctuary sites in cortical stroke

**DOI:** 10.3389/fneur.2025.1606666

**Published:** 2025-09-30

**Authors:** Tharani Thirugnanachandran, Henry Ma, Jason Vuong, Shaloo Singhal, Lee-Anne Slater, Thanh Phan

**Affiliations:** ^1^Stroke and Ageing Research (STAR), School of Clinical Sciences at Monash Health, Monash University, Clayton, Melbourne, VIC, Australia; ^2^Monash Health, Diagnostic Imaging, Monash Health, Clayton, Melbourne, VIC, Australia

**Keywords:** sanctuary sites, leptomeningeal anastomoses, ischemic penumbra, topography, stroke

## Introduction

Sanctuary sites (1) is a term used to describe regions of brain parenchyma with a low probability of infarction compared to other areas, due to the compensatory capacity of leptomeningeal anastomoses (LMA) following vessel occlusion (2, 3). The importance of LMA has been evident since the experiments by Heubner in 1874 (4). Heubner witnessed the filling of the entire arterial tree of the cerebral cortex after injecting colored liquid into a single artery, proximally ligated so there was no connection with the Circle of Willis (CoW) (4). However, the significance of LMA has been questioned. Cohnheim (5) disregarded them and believed in the concept of “end-arteries.” Duret (6) recognized their existence, but he and Charcot (7) questioned their functionality due to their small caliber. Beevor (8) acknowledged their presence anatomically and described variabilities in arterial territories but did not make the connection between the two (9–11). In 1925, Fay (12) attempted to prove Cohnheim's (5) theory of “end-arteries,” but found comparable results to Heubner (4) with mercury injection filling all cortical arteries despite the branches of the CoW being tied (12). The first detailed anatomy of LMA was provided by Vander Eecken and Adams (13) in 1953. Similar observations have been documented by Wollschlaeger and Wollschlaeger (14) and Lazorthes et al. (15). These arterial connections have also been extensively confirmed angiographically (16–23). The small diameter of these vessels and the substantial inter and intra-individual variability may account for the initial lack of recognition (9, 13, 17, 23–26). Current evidence for LMA maintaining the ischemic penumbra and reducing infarct growth is apparent in the extended time window reperfusion trials (27, 28). Collateral status is now recognized as a key determinant of reperfusion and clinical outcome following endovascular clot retrieval (28–31). However, a comprehensive understanding of the anatomy, location and prevalence of leptomeningeal collaterals (9), sanctuary sites (1) and their importance in modifying stroke deficits is still in development.

## The concept of sanctuary sites in ischemic stroke

**Figure 1:**
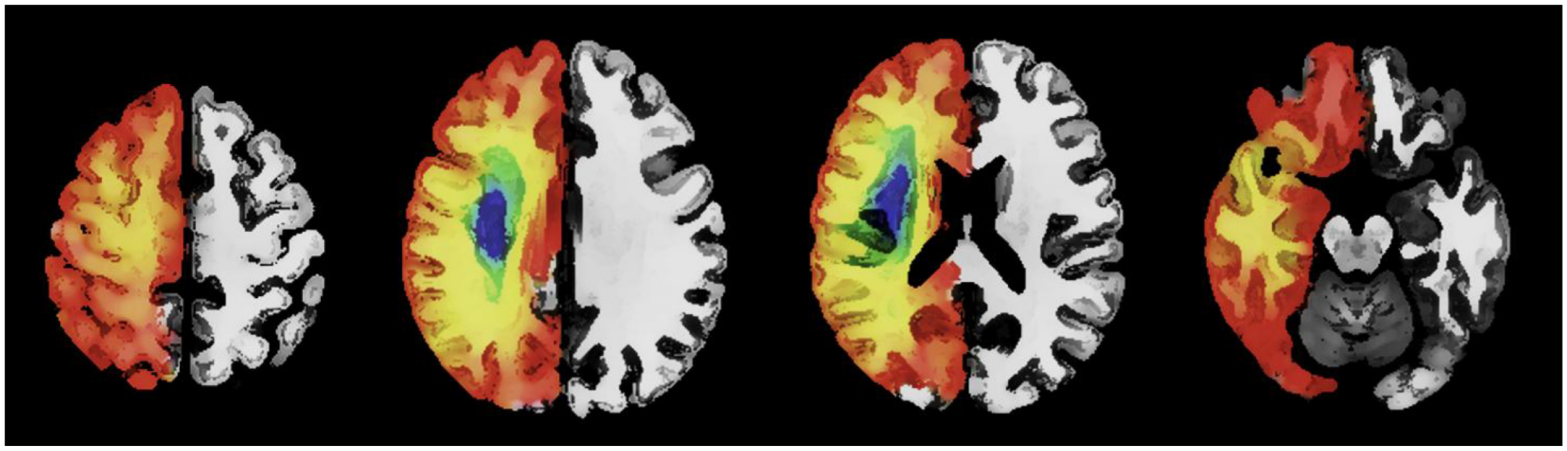
An axial image with a schematic representation of the topography of sanctuary sites created using published digital maps of cortical stroke with documented vessel occlusion. Leptomeningeal collaterals may support blood flow to the superficial compartment to maintain penumbra. Regions (red/orange) have a low probability of infarction and a high probability of sanctuary sites. Regions (blue/green) have a high probability of infarction and a low probability of sanctuary sites.

Sanctuary sites are regions of potentially salvageable penumbral tissue predominantly present within the frontal, parietal and occipital cortex (1). Figure 1 is a schematic representation of sanctuary sites (1) created using published digital maps of cortical stroke with documented vessel occlusion (3, 32–34). Segmented infarcts from T2-weighted magnetic resonance images were averaged to generate a probability of infarction at a voxel level for each arterial territory. The probability of infarction at each voxel (P*i*) for the combined arterial territories was calculated as previously described (1). The probability of sanctuary sites was calculated using the formula probability of sanctuary sites = 1–P*i*. Sanctuary sites were identified as regions with a probability of infarction (P*i*) < 0.1 (1).

Historical data and current works have alluded to the presence of sanctuary sites (1, 3, 13, 17, 33, 35–38). Following proximal MCA occlusion it is well recognized that the highest probability of infarction centers around the striatocapsular region and the centrum semiovale, followed by the insular (24, 33, 35, 37, 39, 40). Compensatory flow from posterior cerebral artery (PCA) and anterior cerebral artery (ACA) to MCA through LMA may enable sparing of cortical regions of the frontal and parietal lobes from infarction (1, 13, 17, 33, 35–38), following reperfusion. Although we acknowledge inter-individual variability in LMA, we propose that knowledge of the detailed anatomy of the anastomoses as described by Vander Eecken and Adams (13), and others (14–16, 26) may be useful to increase our understanding of the potential locations of sanctuary sites (1, 3, 32–34). Table 1 describes the possible locations of sanctuary sites using data from previously published digital atlas of probability of infarction (3, 32–34) and the sanctuary sites map (1). To assist with anatomic interpretation, a database Talairach Daemon was used to relate the voxel coordinate in the X, Y, and Z planes (available at http://www.talairach.org/daemon.html).

**Table 1:** Location of possible sanctuary sites (regions of low probability of infarction due to blood supply from leptomeningeal anastomoses).

**Sanctuary site**				**Leptomeningeal collateral**								
**Gyrus**	**Voxel coordinates (X, Y, Z)**	**Cortex**	**Function**	**ACA**	**Probability of infarction**	**Probability of sanctuary sites**	**MCA**	**Probability of infarction**	**Probability of sanctuary sites**	**PCA**	**Probability of infarction**	**Probability of sanctuary sites**
Gyrus rectus	7.6, 36.0, −17.9	Orbitofrontal cortex	Unclear function	Medial Orbitofrontal artery	0.02	0.98	Lateral Orbitofrontal artery	NA	NA	NA	NA	NA
Orbitofrontal gyrus	17.2, 47.8, −14.2	Orbitofrontal cortex	Cognitive process of decision making	Medial Orbitofrontal artery	0.02	0.98	Lateral Orbitofrontal artery	NA	NA	NA	NA	NA
Middle frontal gyrus	36.3, 33.0, 33.7	Premotor cortex	Motor planning	Posterior internal frontal artery	0.05	0.95	Precentral artery	0.05	0.95	NA	NA	NA
Precentral gyrus	40.0, −8.3, 52.1	Primary motor cortex	Motor	Paracentral artery	N/A	N/A	Central artery	0.05	0.95	NA	NA	NA
Postcentral gyrus	40.0, −25.2, 52.9	Primary sensory cortex	Sensory	Superior parietal artery	NA	NA	Anterior parietal artery	0.02	0.98	NA	NA	NA
Superior parietal lobule	25.3, −59.1, 62.5	Superior parietal lobule	Attention and Spatial orientation	Superior parietal artery	NA	NA	Anterior parietal artery	0.02	0.98	NA	NA	NA
Inferior parietal lobule	45.2, −46.6, 49.2	Somatosensory cortex	Sensory processing and integration	NA	NA	NA	Posterior parietal artery	0.03	0.97	Parieto-occipital artery	NA	NA
Supramarginal gyrus	56.2, −31.9, 34.4	Somatosensory cortex	Phonological processing	NA	NA	NA	Posterior parietal artery	0.07	0.93	Parieto-occipital artery	NA	NA
Angular gyrus	44.4, −59.9, 38.9			NA	NA	NA	Angular artery	0.05	0.95	Parieto-occipital artery	NA	NA
Paracentral lobule	3.9, −37.0, 68.3	Somatosensory association cortex	Somatosensory function contralateral lower limb	Paracentral artery	0.01	0.99	Central artery	NA	NA	NA	NA	NA
Posterior cingulate gyrus	6.8, −42.2, 21.9	Paralimbic cortical structure	Default mode network	Posterior pericallosal artery	NA	NA	NA	NA	NA	Parieto-occipital artery (callosal branch)	0.01	0.99
**Gyrus**	**Voxel coordinates (X, Y, Z)**	**Cortex**	**Function**	**ACA**	**Probability of infarction**	**Probability of sanctuary sites**	**MCA**	**Probability of infarction**	**Probability of sanctuary sites**	**PCA**	**Probability of infarction**	**Probability of sanctuary sites**
Precuneus	9.0, −56.2, 44.0	Superior parietal lobule	Episodic memory, default mode network (resting consciousness)	Posterior pericallosal artery or precuneal artery	0.01	0.99	NA	NA	NA	Parieto-occipital artery (callosal branch)	NA	NA
Cuneus	12.7, −79.8, 28.5	Visual cortex	Superior optic radiations	Posterior pericallosal artery precuneal artery	NA	NA	NA	NA	NA	Parieto-occipital artery (callosal branch)	0.02	0.98
Superior occipital gyrus	23.1, −80.5, 30.7	Occipital cortex	Visual recognition of objects	NA	NA	NA	Posterior temporal artery	0.02	0.98	Parieto-occipital artery	NA	NA
Middle occipital gyrus	36.3, −79.8, 18.9	Occipital cortex		NA	NA	NA	Posterior temporal artery	0.04	0.96	Parieto-occipital artery	NA	NA

Voxel coordinates in the X, Y, and Z planes represent their location on a standard brain template.

## Vascular anatomy of collateral systems

During the embryo and fetal stages of ontogenesis, the vascular supply for the brain is exclusively from the internal carotid artery (ICA) (41). Pial collaterals develop in early fetal life, connecting branches of the rostral and caudal trunk of the ICA. The rostral trunk of the ICA becomes the ACA medially and MCA laterally and the caudal trunk becomes the PCA (41). The anterior communicating artery (AcomA) forms an anastomotic connection between the two ACA and the posterior communicating artery (PcomA) joins the ICA to the PCA. Following progressive atrophy of the PcomA the vertebral system develops (41). In proximal vessel occlusion, the CoW redistributes blood from posterior to anterior circulation via the PcomA or between hemispheres via the AcomA. In imaging studies, a complete configuration of the circle is reported in <42% of people (42, 43) and its ability to rescue in occlusions distal to CoW via the PcomA has been previously overstated (44). Following MCA occlusion, the CoW is unable to salvage penumbra (2). Perfusion to the MCA territory is instead maintained via recruitment of LMA (2). These arteriole-arteriole anastomoses connect select distal branches of the ACA, MCA, and PCA (13, 21, 25) as either end-to-end or candelabra anastomoses (13, 21). Luminal size and number are important factors which determine the ability of LMA to maintain cerebral perfusion (45). In agreement with Heubner (4), Van der Zwan and Hillen (10, 11) reported diameters as large as 1 mm. A computer model of the cerebral circulation has also shown that similar sizes of the LMA were necessary to keep cerebral blood flow above 30% (2). Furthermore, animal models have also shown that LMAs which connect branches of ACA and MCA, have larger luminal size and reduced basal tone and myogenic reactivity compared to pial arterioles which do not (46).

## Location and impact of sanctuary sites

### LMA between ACA and MCA

Most LMA occur between the ACA and the MCA (9, 13, 18, 21–23). These anastomoses are crucial to supporting the metabolic needs of important motor and somatosensory areas (see Table 1) (1, 3). A frequent occurrence is the presence of double anastomotic channels in the pre-central, central and post-central regions (14). The posterior internal frontal artery, a branch of the callosomarginal artery forms end-end anastomosis in the pre-central sulcus with the precentral artery (13, 16, 17) (from the MCA) to supply the anterior border of the precentral gyrus (1, 14). In the central sulcus, the paracentral artery and the central artery (13, 16, 17) form connections to supply the posterior border of the precentral gyrus (14), the anterior border of the postcentral gyrus (1, 14) and the paracentral lobule (3, 47, 48). Anastomoses of the superior parietal branch (also known as precuneal branch) with the anterior parietal artery (13, 14, 16, 17) lie within the postcentral sulcus to supply the posterior border of the postcentral gyrus (1) and superior parietal lobule (1) above the intraparietal sulcus. In ACA stroke, these anastomoses limit ACA infarct topography and result in sparing of M1 fiber tracts (3, 48) which may impact motor outcome. These LMA may also be important following MCA occlusion (49). Inferomedially, sanctuary sites exist in the orbital gyrus and gyrus rectus (1, 3, 36) due to anastomoses from the medial and lateral orbitofrontal arteries (13, 14). The low probability of infarction of the rostral aspect of the superior frontal gyrus (3) may be due to compensation from fine transverse or oblique connections between the frontopolar branch (of the ACA) (16) with the orbito-frontalis branch (of the MCA) (13, 16). Following occlusion of the MCA (33) or ACA, (3) the low probability of infarction of the middle frontal gyrus (3, 33) (see Table 1) is likely due to anastomoses between the anterior and middle internal frontal arteries (16, 21, 26) (from the ACA) and the superior branches of the orbitofrontal artery (from the MCA) (9, 13, 16, 21, 26) which lie in the superior frontal sulcus.

### LMA between ACA and PCA

Medially, LMA between the terminal branches of the pericallosal artery (14) or precuneal artery (13, 14, 16, 17) (from the ACA) and the posterior callosal branch of the parieto-occipital artery (from the PCA) (13, 14, 16, 17, 21) may account for the low probability of infarction in the precuneus (1, 3) and posterior cingulate gyrus (1, 3) following ACA stroke and the cuneus (1, 34, 50) and splenium (34, 51) following PCA stroke.

### LMA between MCA and PCA

End to end anastomosis between the posterior parietal (16), posterior temporal (13) or angular artery (13, 16, 17, 21) (from the MCA) with the parieto-occipital artery (13, 16, 17, 21) (from the PCA) lie within the inferior portion of the intraparietal sulcus or superior portion on the parieto-occipital sulcus (9, 13). Following occlusion of the MCA, they may account for the low probability of infarction seen in the supramarginal and angular gyrus (1, 33) and following PCA occlusion they may explain the presence of sanctuary sites in the superior and middle occipital gyrus (1, 50) and the inferior parietal lobule (1) (Table 1).

### LMA between ACA and contralateral ACA

Finally, in almost two thirds of cases (52), the distal branches of the ACA extend to the medial surface of the contralateral hemisphere and can form anastomoses with branches of the pericallosal (16, 21) and callosomarginal arteries (13, 21) including the precuneal and paracentral arteries (9, 13). These branches may further support blood flow to the precuneus, paracentral lobule, posterior cingulate gyrus (1, 3) and splenium (52).

## Haemodynamic factors affecting LMA

Based on simulation studies, in the absence of vessel occlusion, low flow within LMA occurs due to the lack of pressure difference between arterial territories. Following obstruction of an artery, the subsequent drop in blood flow and a fall in pressure in downstream vessels results in reversal in flow direction and hemodynamic recruitment of LMA surrounding the occluded vessel (53). This retrograde flow produces an increase in blood flow through LMA via an increase in lumen diameter and a fall in vascular resistance. This helps to maintain cerebral perfusion and support the penumbra until recanalization and reperfusion (53). LMA give rise to penetrating arterioles which pierce the cortical surface and enter the brain substance to supply the capillaries in the microvascular sub-surface bed (26, 54, 55). Each penetrating arteriole forms a vascular unit. As these arterioles lack anastomoses (56), they can leave subcortical structures vulnerable to profound ischemia if they are occluded (54). In animal models, flow reversal in LMA and active dilation of penetrating arterioles lying close to an LMA restores blood flow to regions of ischemia following MCA occlusion (55). In contrast, regions further away from LMA are less able to provide compensatory flow, resulting stasis of blood in penetrating arterioles with an increased risk of infarction (54). A combination of genetic and vascular risk factors likely accounts for the significant inter-individual variation seen in leptomeningeal collaterals. We will review these in the following paragraph.

## Genetic and vascular risk factors affecting LMA

In mice, genetic background is a major determinant of this variation, with approximately 80% found localized to the Rabep2 gene (57). Collaterogenesis, can occur secondary to hypoxia and vessel occlusion (58) and was abolished in mice lacking Rabep2 gene (57). Lower arterial oxygen levels in pial watershed areas, is thought to stimulate collaterogenesis through increased expression of Rabep2 gene and increased signaling of vascular endothelial growth factor (VEGF-A) (57, 59). It is unclear at present whether similar genetic polymorphisms will be discovered which account for the variation in collaterals seen in humans.

Several vascular risk factors have been shown to influence the development of collaterals. Age is the most important risk factor for ischemic stroke (60), and its influence on collaterals has been studied in both humans (61–64) and animal models (65, 66). In mice models, with increasing age, a process known as collateral “rarefaction” can occur (65). This results in a reduction in the number and luminal diameter of collaterals leading to increased vascular resistance. Additionally, animal models have shown that aging is believed to impair the capacity of pial arteries to dilate (65, 66). Collateral rarefaction can also occur in the presence of vascular endothelial dysfunction and cardiovascular risk factors (66, 67). Hypertension, has been shown to reduce the development and compensatory capacity of collaterals following vessel occlusion in both animal models (46, 66, 68, 69) and humans (64, 70–72). In spontaneously hypertensive rat models, LMA responded with increased myogenic vasoconstriction in response to elevated pressure (46, 69). The resulting vascular dysfunction and vasoconstriction compromises collateral flow and can increase susceptibility to ischemic injury. An important system in the pathogenesis of hypertension is the renin-angiotensin system. Treatment with an angiotensin converting enzyme inhibitor and subsequent lowering of ANG II levels prevented vasoconstriction of LMA and reversed vascular dysfunction, independent of blood pressure lowering (73).

Hyperlipidemia through the formation of atherosclerosis, is also believed to stimulate ischemic preconditioning and collaterogenesis (74). Favorable collaterals have been associated with hyperlipidemia in humans (61). In mouse models, statins have been shown to upregulate endothelial nitric oxide synthetase resulting in increased cerebral blood flow and reduction in infarct size (75–77). However, the presence of prior statin use has shown conflicting results in humans, with some studies suggesting statin treatment prior to stroke resulted in the presence of higher collaterals grades on angiography (78), even in those with cardioembolic stroke (79). Whilst other studies have shown poorer collaterals in stroke patients with prior-stroke statin use (61, 62).

Poor collateral status and faster evolution from penumbra to infarct core has also been associated with acute and chronic hyperglycemia (64, 71, 72, 80, 81), in patients with type 2 diabetes (82, 83), and in smokers (61). Improving our understanding of clinical factors which impact collaterogenesis or collateral rarefaction may enable us to develop effective therapies in the future.

## Collateral therapeutics

Despite a plethora of studies investigating neuroprotection agents in stroke, successful augmentation of collateral hemodynamics following vessel occlusion has provided mixed results, with some interventions showing limited success (84, 85). Early trials of head positioning to improve cerebral perfusion in ischaemic stroke were neutral, but showed that the fully supine position was safe (86). More recently pre-clinical trials (87) and clinical trials (88, 89) investigating head positioning in large artery occlusion have shown more promise. In a pre-clinical randomized trial, positioning with head down using a 15° tilt resulted in increased cerebral blood flow, reduced infarct volume and improved clinical outcome (87) in rodent models with middle cerebral artery occlusion. Unsurprisingly this benefit was greater in subjects with good collaterals, but even a mild improvement was seen in cases with poor collaterals (90). In humans, studies have shown head-down positioning (−20^o^ Trendelenburg position) was well tolerated and resulted in a modest improvement in cerebral perfusion on imaging (91). A randomized multicenter trial of prolonged head down positioning in patients with large artery atherosclerosis of the anterior circulation not suitable for reperfusion therapies, also showed that it was safe but was statistically neutral for the primary outcome of 90 day functional independence (modified Rankin scale 0–2) **(****88****)**. In contrast, head positioning when used prior to endovascular clot retrieval in patients with large vessel occlusion, has been shown in a small randomized clinical trial to reduce neurological deterioration, defined as an National Institutes of Health Stroke Scale (NIHSS) of 2 or more (89).

Pre-clinical studies in animal models have shown that induced hypertension can improve cerebral blood flow through collaterals and reduce infarct volume (92, 93). In a multicenter randomized clinical trial, therapeutic induced hypertension using phenylephrine in patients with non-cardioembolic stroke ineligible for reperfusion therapies was associated with early neurological improvement and functional independence at 90 days (94). Blood pressure augmentation may be beneficial in selected patients (94) but further randomized controlled trials are needed to define its safety profile and optimal use.

## Implications and future directions

The presence of LMA between cortical branches of the ACA and MCA may support blood flow to the superficial compartment to maintain penumbra (see Figure 1). Therefore, the concept of sanctuary sites (1) may provide a framework for identifying patients with a large core (95–98) who have the ability to reach a good functional outcome following treatment with clot retrieval. Imaging studies with MRI suggest that in some cases penumbra can exist up to 48 h (99). Development of adjuvant medical therapies which augment blood flow through LMA (100) may enable extension of current time windows for reperfusion therapies and allow successful treatment of patients who require long distance transfer to a comprehensive stroke center for thrombectomy (101). Pharmacological augmentation of blood flow through established LMA may also be sufficient to improve clinical outcome in cases of large and medium vessel occlusion not suitable for endovascular clot retrieval (100, 102–105) or where thrombolysis is contraindicated (100).

## Conclusion

We have identified regions with a low probability of infarction due to the functionality of LMA. In the modern era of reperfusion therapy, knowledge of the locations of sanctuary sites may help guide therapeutic management. Augmenting collaterals to support blood flow to sanctuary sites may be beneficial when used in conjunction with endovascular clot retrieval, especially if there are delays to treatment.
